# Dual microbial inoculation for tripartite benefits: soybean biomass enhancement, root rot control and chlorothalonil residue reduction

**DOI:** 10.3389/fpls.2025.1585035

**Published:** 2025-08-08

**Authors:** Weiguang Jie, Min Zhang, Yiwen Tan, Haobo Yang, Wenkai Wang, Lianbao Kan

**Affiliations:** ^1^ Ministry of Education & Heilongjiang Provincial Key Laboratory of Plant Genetic Engineering and Biological Fermentation Engineering for Cold Region, Engineering Research Center of Agricultural Microbiology Technology, College of Heilongjiang Province & School of Life Sciences, Heilongjiang University, Harbin, China; ^2^ Key Laboratory of Microbiology, College of Heilongjiang Province & School of Life Sciences, Heilongjiang University, Harbin, China; ^3^ School of Food Engineering, Heilongjiang East University, Harbin, China

**Keywords:** *Rhizophagus intraradices*, *Acinetobacter calcoaceticus*, AMF colonization rate, bacterial community, soybean rhizosphere soil

## Abstract

As global agriculture shifts toward an ecological civilization model, microbial fertilizers have emerged as a new strategy to promote plant growth and enhance soil fertility. In this study, the effects of *Rhizophagus intraradices* and *Acinetobacter calcoaceticus* on soybean biomass, root rot disease index, chlorothalonil residue in soybean rhizosphere soil and grains, and the composition of the bacterial community in the rhizosphere soil were investigated through pot experiments. Soybean biomass, chlorothalonil residue and bacterial community were analyzed by direct measurement method, gas chromatographic method and high throughput sequencing, respectively. The findings demonstrated that in the *R. intraradices* and *A. calcoaceticus* inoculation group, AMF spore density and colonization rate increased by 116.42% and 139.17%, respectively, compared to the control group. Microbial inoculum significantly enhanced the soybean biomass. Compared with the control group, the hundred-grain weight in the *R. intraradices* and *A. calcoaceticus* inoculation group increased by 35.46%. The disease index of soybean root rot decreased by 77.78% in the *R. intraradices* and *A. calcoaceticus* inoculation group relative to the control group. Furthermore, compared with the chlorothalonil-treated group, the chlorothalonil residue in both the rhizosphere soil and soybean grains in the chlorothalonil-treated and inoculated with *R. intraradices* and *A. calcoaceticus* group decreased by 80.02% and 81.65%, respectively. Additionally, microbial inoculum and chlorothalonil application exerted substantial effects on the composition of the bacterial community. Specifically, co-inoculation with *R. intraradices* and *A. calcoaceticus* led to an increase in the relative abundance of Acidobacteriota and Patescibacteria in the rhizosphere soil. Conversely, chlorothalonil application resulted in a reduction in the relative abundance of these bacterial taxa. The primary objective of this study was to provide theoretical support for the application of microbial inoculum as a strategy to mitigate soybean root rot, enhance growth, and reduce pesticide residue, thereby contributing to sustainable agricultural practices.

## Introduction

Soybean (*Glycine max* L.), a globally significant crop, is highly valued for its rich content of protein, oil, vitamins, and other essential nutrients, serving as a primary source for food, feed, and biodiesel production (Chen et al., 2025; Coleman et al., 2021). In China’s major soybean-producing regions (Northeast and Huang-Huai-Hai), soybean cultivation plays a critical dual role in sustainable agriculture. It not only ensures food security and stabilizes farm incomes but also sustains agroecosystem balance by leveraging its symbiotic relationship with *Rhizobia* for nitrogen fixation. This ecological mechanism substantially lowers reliance on synthetic fertilizers and simultaneously improves soil carbon sequestration capacity (Ngosong et al., 2022). Collectively, these functions underscore soybean’s dual identity as a “green nitrogen factory” and a soil health protector, demonstrating its indispensable contribution to environmentally conscious farming practices. However, in China, the combination of limited arable land resources and the vulnerability of soybean to root rot has led to a decline in both yield and quality (Hartman et al., 2016; Okello et al., 2020; Williamson-Benavides and Dhingra, 2021). To address this issue, chemical pesticides have been widely employed for the management of root rot. Among these, chlorothalonil, a high-efficiency, low-toxicity, broad-spectrum fungicide, exerts its antifungal effects by disrupting the activity of glycerol 3-phosphate dehydrogenase in fungal cells, leading to impaired metabolic function and eventual cell death (Thomidis and Michailidis, 2002). Consequently, chlorothalonil has been extensively utilized in agricultural practices for its efficacy in controlling fungal diseases. Chlorothalonil’s long-term application has led to persistent soil residues in Heilongjiang Province. Although effective as a broad-spectrum fungicide, chlorothalonil exhibits high environmental persistence, resulting in gradual accumulation of potentially toxic residues that threaten soil ecosystem stability and pose long-term risks to human health (Tao et al., 2024; Karabörklü et al., 2018). In recent years, advances in agricultural biological control technologies have sparked considerable interest in the application of microorganisms for disease management. Biological control strategies not only mitigate plant diseases but also enhance crop productivity, safeguard environmental sustainability, and promote soil health.

Arbuscular mycorrhizal fungi (AMF) can form a symbiotic relationship with more than two thirds of terrestrial plants (Duan et al., 2024; Farhaoui et al., 2025; Wang et al., 2022). After the symbiosis between AMF and plants, the extrarhizal hyphal network of AMF will expand the absorption area of plant roots in the soil, and the hyphae can penetrate into the areas that cannot be accessed by plant roots, increasing the uptake and utilization of soil nutrients by host plants (Duan et al., 2025; Wipf et al., 2019). At the same time, plants can provide energy and carbon sources for AMF through photosynthesis (Shi et al., 2023). This symbiotic relationship allows plants to better adapt to the soil environment and promote plant growth. Parihar et al. (2020) demonstrated that AMF (*Funneliformis mosseae* and *Rhizophagus intraradices*) inoculation significantly increased pea’s antioxidant enzyme activity, improved nutrient balance, and increased pea yield. In addition, AMF can significantly improve plant disease resistance, including resistance to pathogenic bacterial attacks. Studies have shown that AMF can induce disease resistance in plants by regulating the types and quantities of secondary metabolites in the physiological metabolism of host plants (Farhaoui et al., 2025; Li et al., 2010). *Acinetobacter calcoaceticus* is a phosphorus solubilizing bacterium that converts soil insoluble organic phosphorus into soluble inorganic phosphorus, thereby improving the uptake and utilization of phosphorus by AMF and plants (Peix et al., 2009; Srivastava and Srivastava, 2006). At the same time, *A. calcoaceticus* also has the ability of nitrogen fixation, phosphorus and potassium solution, IAA production, and siderophore, which can promote plant growth and development (De La Torre-Ruiz et al., 2016; Prajapati et al., 2022). Laha et al. (2024) studies have shown that inoculation with *A. calcoaceticus* promote the growth and development of Lentil and increase its production. Therefore, the combined application of AMF and *A. calcoaceticus* can further promote the growth and development of plants, improve disease resistance.

A comprehensive understanding of the effects of microbial inoculum on soybean biomass, chlorothalonil residue in soybean rhizosphere soil and grains, and the composition of the bacterial community in the rhizosphere soil is critically important. The study was guided by the following hypotheses: (1) *R. intraradices* and *A. calcoaceticus* significantly enhance the colonization rate and spore density of AMF, increase the number of root nodules, and promote soybean biomass. (2) *R. intraradices* and *A. calcoaceticus* reduce chlorothalonil residue in both the rhizosphere soil and soybean grains. (3) The application of microbial inoculum and chlorothalonil alters the composition of the bacterial community in the soybean rhizosphere soil.

## Materials and methods

### Experimental materials

The root rot-susceptible soybean cultivar Heinong 48, obtained from Heilongjiang Academy of Agricultural Sciences. The soybean cultivar has been widely planted in the Heilongjiang Province of China.

*Rhizophagus intraradices* was isolated from the rhizosphere soil of soybean fields in Heilongjiang Province by our research team. *R. intraradices* was propagated in a pot culture with alfalfa plants grown in sterilized vermiculite, river sand, and soil (3: 2: 5, *v/v/v*) for approximately 5 months. Post-harvest analysis revealed an AMF colonization rate of 94.2% and a spore density of 498 spores per 10 grams of air-dried soil.

*Acinetobacter calcoaceticus* was also isolated from the rhizosphere soil of soybean fields in Heilongjiang Province. *A. calcoaceticus* was cultured overnight in LB liquid medium, centrifuged (5,000 × g, 5 min), and resuspended in a sterile aqueous solution to achieve a final concentration of 1 × 10^7^ CFU/mL.

The broad-spectrum protective fungicide chlorothalonil, chemically known as tetrachlorodibenzonitrile (C_8_Cl_4_N_2_), was used in this study.

### Experimental design

The experiment was conducted using potted plants. Eight treatment groups were established: blank control (CK); chlorothalonil (C); *R. intraradices* (R); *A. calcoaceticus* (A); *R. intraradices* and *A. calcoaceticus* (RA); chlorothalonil and *R. intraradices* (CR); chlorothalonil and *A. calcoaceticus* (CA); chlorothalonil, *R. intraradices*, and *A. calcoaceticus* (CRA). Nine replicates were prepared in each treatment group. To mitigate potential marginal effects, pots of identical size were arranged around the experimental pots as a protective barrier.

Each pot was filled with 12 kg of soil collected from a soybean field located in Pingfang District, Harbin, Heilongjiang Province (45°66’ N, 126°61’ E). The soil type was typical black soil (Mollisols, classified by the USDA Soil Taxonomy). Initial soil characterization indicated: pH 7.16, organic matter 26.95 g/kg, total nitrogen 1.28 g/kg, total phosphorus 0.91 g/kg, total potassium 23.86 g/kg, loam particle content 34.13%, sand particle content 43.12%, and clay particle content 22.75%. *R. intraradices* inoculum (90 g) was applied to the corresponding treatment group. A soil thickness of 1-2 cm was evenly spread on inocula, and then the soybean seeds were evenly spread on the soil layer. Finally, a soil thickness of 1-2 cm was evenly spread on the soybean seeds. Six soybean seeds were sown in each pot. *A. calcoaceticus* inoculum (5 mL) was inoculated into the soil via root irrigation for each soybean seed in the corresponding treatment group. At 60 and 90 days after soybean emergence, a 5 mg/mL solution of chlorothalonil was evenly applied to the corresponding rhizosphere soil, and the amount of spraying was 21 mL per pot.

### Samples collection

Rhizosphere soil samples were randomly collected at 120 days after soybean emergence. During this period, the dry matter content of soybean grains reached its peak, and the physiological indicators of the plants were significantly positively correlated with the final yield. These samples were divided into two portions: one for the quantification of AMF spore density and chlorothalonil residue, and the other frozen at -80°C for subsequent DNA extraction.

Three soybean plants were randomly harvested from each treatment at 120 days post-emergence to assess the following parameters: plant height, stem diameter, root length, hundred-grain weight, pod number, aboveground dry weight, underground dry weight, aboveground fresh weight, underground fresh weight, and nodule number. Detailed measurement procedures followed the methodologies outlined by Rotundo et al. (2012).

### Analysis of AMF spore density, AMF colonization rate, disease index of soybean root rot, and total bacterial colonies

AMF spore density in the rhizosphere soil was determined using a wet sieve decanting and sucrose centrifugation method, as described by Zhou et al. (2011).

AMF colonization rate was determined using the alkali separation-acid fuchsin method according to Zhou et al. (2011) Additionally, the disease index of soybean root rot was evaluated according to the method described by Cui et al. (2018).

Total bacterial colonies in the rhizosphere soil were quantified using 10 g of soil, following the protocol detailed by Jie et al. (2023).

### Determination of chlorothalonil residue

The soybean grains were thoroughly ground, and the soil samples were sieved through a 40-mesh sieve for subsequent analysis. Precisely 5.0 g of each sample was weighed and homogenized with 10 mL of acetonitrile using a homogenizer for 2 min. The homogenate was filtered through filter paper, and the filtrate was collected into a 100 mL graduated cylinder containing 1 g of NaCl. A 5 mL aliquot of the filtrate was transferred to a separate container, sealed, and vigorously shaken for 1 min. The mixture was then allowed to stand at room temperature for 30 min to facilitate phase separation between acetonitrile and water. A 10.00 mL portion of the acetonitrile phase was transferred into a beaker and placed in an 80°C water bath. Nitrogen gas was gently introduced to evaporate the solution to near dryness. Subsequently, 2 mL of n-hexane was added, and the mixture was covered with aluminum foil for purification. A Florisil column was pre-conditioned with 5 mL of acetone + n-hexane (1:9, v/v) followed by 5 mL of n-hexane. When the solvent reached the surface of the adsorbent layer, the purification solution was immediately added, and the eluate was collected in a graduated centrifuge tube. The column was rinsed twice with 5 mL of acetone + n-hexane (1:9, v/v). The eluate was then concentrated to less than 5 mL using a nitrogen evaporator at 50°C. The volume was adjusted to 5 mL with n-hexane, vortex-mixed, and transferred to the autosampler for analysis. An Agilent HPGC7890A gas chromatograph (Agilent Technologies Inc., CA, USA) was employed for the analysis. The chromatographic conditions were as follows: inlet temperature, 200°C; detector temperature, 320°C; column temperature programmed at 150°C (hold for 2 min), ramped at 6°C/min to 270°C (hold for 18 min); carrier gas, high-purity nitrogen (purity ≥ 99.999%) at a flow rate of 1 mL/min; split injection with a ratio of 10:1.

Chlorothalonil residue calculation:

Chlorothalonil in the sample to be tested is calculated by mass fraction ω, mg/kg:


ω=ρ×(V1×A×V3)/(V2×AS×m)

In the formula: ρ—Mass concentration of chlorothalonil standard (mg/L);

A— Peak area of chlorothalonil in the sample;

AS—Peak area of the chlorothalonil standard;

V1— Total volume of the extraction solvent (mL);

V2—Volume of the extracted solution aspirated for detection (mL);

V3—Volume of the sample solution (mL);

m—Mass of the sample (g).

### DNA extraction, sequencing and data preprocessing

DNA was extracted from 0.5 g of soybean rhizosphere soil using the PowerSoil DNA Isolation Kit (MOBIO Laboratories Inc., Carlsbad, CA, USA), adhering to the manufacturer’s instructions. Bacterial diversity was assessed via 16S rRNA gene amplicon sequencing on a PacBio platform (Majorbio Biopharm Technology Co., Ltd., Shanghai, China). The V3-V4 region of the 16S rRNA gene was amplified using primer 338F (5′-ACTCCTACGGGAGGCAGCAG-3′) and 806R (5′-GGACTACHVGGGTWTCTAAT-3′) (Wang et al., 2021). PCR amplification was conducted in a 20 μL reaction volume containing 10 μL of 2× Pro Taq, 0.8 μL of each primer (5 μM), 200 ng of DNA template, and ddH_2_O. The thermal cycling protocol included initial denaturation at 95°C for 3 min, followed by 30 cycles of denaturation at 95°C for 30 s, annealing at 50°C for 30 s, extension at 72°C for 45 s, and a final elongation at 72°C for 10 min. PCR products were analyzed by 2.0% agarose gel electrophoresis, purified using the AxyPrep DNA Gel Extraction Kit (Axygen Biosciences, Union City, CA, USA), and quantified using QuantiFluor™-ST (Promega, USA). Equal concentrations of PCR products were pooled for library construction and sequencing.

Raw sequences were processed and filtered using FASTP v0.19.6 (Chen et al., 2018) and FLASH v1.2.11 (Magoč and Salzberg, 2011) software. Operational taxonomic units (OTUs) were clustered at 97% similarity using USEARCH v11 (Tarek et al., 2015) software. Taxonomic classification and identification of OTUs were performed using the BLAST (Edgar, 2010) algorithm against the GenBank database, and bacterial species were annotated via the SILVA database v138. The raw sequencing data have been deposited in the NCBI (National Center for Biotechnology Information) database under BioProject accession number PRJNA1228313.

### Statistical analyses

Significant differences between treatments were evaluated using Duncan’s multiple range test (honestly significant differences, HSD) at a significance level of *P*< 0.05, conducted with SPSS 27.0 software (SPSS Inc., Chicago, IL, USA). Data visualization was performed using Origin 2019b. The richness and diversity of the microbial community were assessed using the Ace index (Hughes et al., 2001), Chao1 index (Chao et al., 2005), Shannon index (Shannon et al., 2003), Simpson index (Simpson, 1949), and Good’s Coverage index (Rodrigues et al., 2014), all of which were calculated using MOTHUR v1.30 (Schloss et al., 2009). VENN diagrams were generated to illustrate the distribution of overlapping and unique (OTUs) at a 97% sequence similarity threshold (Edgar, 2013; Stackebrandt and Goebel, 1994). Beta-diversity was evaluated using Bray-Curtis dissimilarity metrics and represented through Principal Coordinate Analysis (PCoA). Bacterial community composition at the phylum and genus levels was represented using histograms, and relative abundance differences at the genus level were illustrated using a heatmap generated with the R v3.3.1 pheatmap 1.0.8 package (Team, 2006).

## Results

### Effects of microbial inoculum and chlorothalonil on the AMF spore density and AMF colonization rate

**Figure 1:**
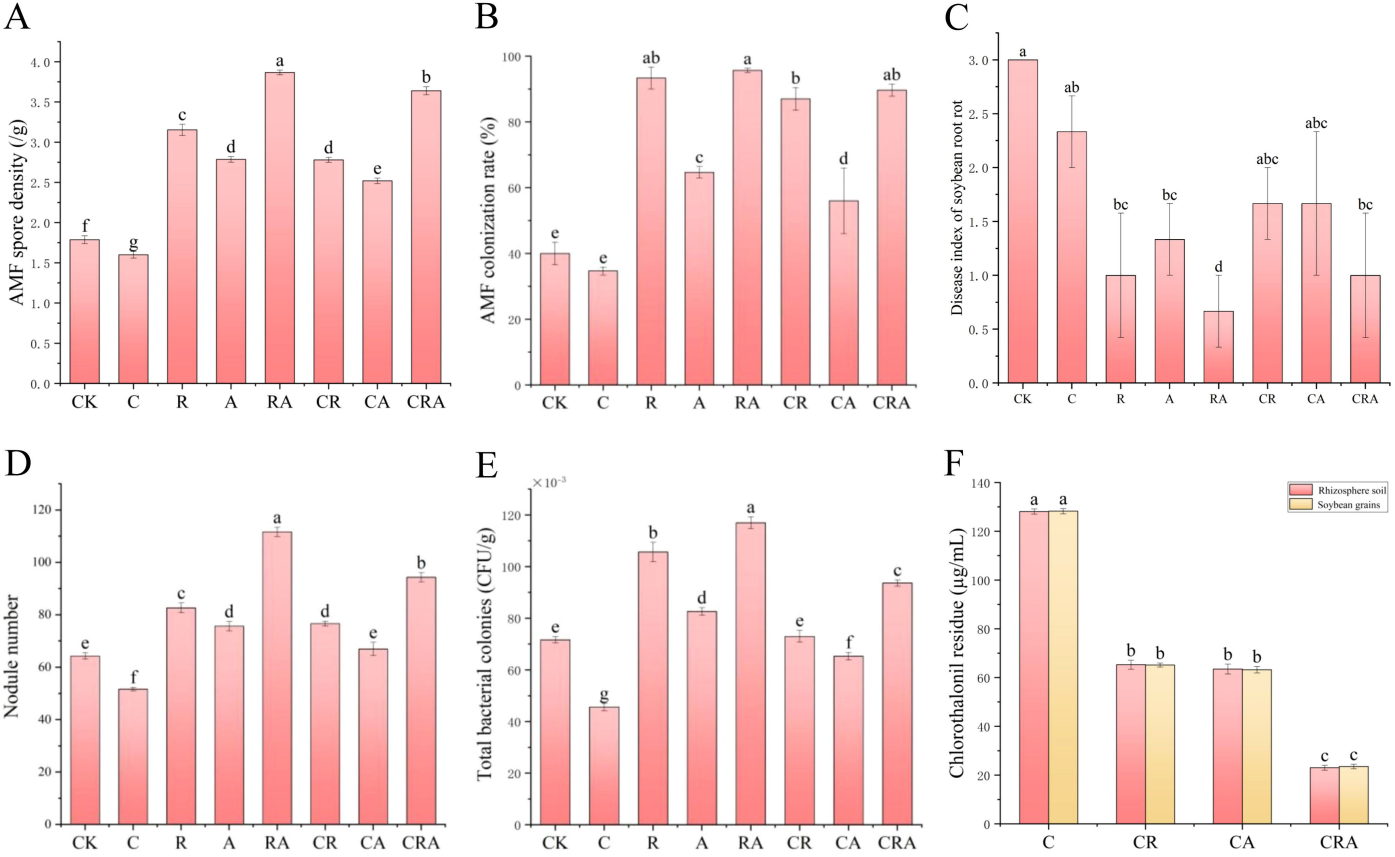
Effects of different treatments on **(A)** AMF spore density, **(B)** AMF colonization rate, **(C)** disease index of soybean root rot, **(D)** nodule number, **(E)** total bacterial colonies, and **(F)** chlorothalonil residue. CK, blank control; C, chlorothalonil; R, *R. intraradices*; A, *A. calcoaceticus*; RA, *R. intraradices* and *A. calcoaceticus*; CR, chlorothalonil and *R. intraradices*; CA, chlorothalonil and *A. calcoaceticus*; CRA, chlorothalonil, *R. intraradices* and *A. calcoaceticus*.

As shown in **Figure 1A**, inoculation with *R. intraradices* significantly increased the AMF spore density in the rhizosphere soil of soybean compared to the control group. Additionally, the AMF spore density in the group inoculated with both *R. intraradices* and *A. calcoaceticus* was 22.86% higher than in the group inoculated with *R. intraradices*, indicating that *A. calcoaceticus* promote the growth and reproduction of AMF spores. In contrast, the AMF spore density in the chlorothalonil-treated group decreased by 10.61% relative to the control group, demonstrating that chlorothalonil inhibits the growth and reproduction of AMF spores in the rhizosphere soil. Notably, under chlorothalonil application, the AMF spore density in the microbial inoculum group was significantly higher than in the non-inoculum group, illustrating that microbial inoculum not only enhances AMF spore density but also mitigates the inhibitory effects of chlorothalonil on AMF spore growth and reproduction.

As illustrated in **Figure 1B**, a significant increase in the AMF colonization rate was observed in soybean roots treated with microbial inoculum compared to the control group, demonstrating that microbial inoculum enhances AMF colonization rate. Conversely, the AMF colonization rate in soybean roots decreased by 12.50% in the chlorothalonil-treated group relative to the control group, indicating an inhibitory effect of chlorothalonil on AMF colonization rate. However, in the group treated with both chlorothalonil and the combined inoculum of *R. intraradices* and *A. calcoaceticus*, the AMF colonization rate increased by 157.14% compared to the chlorothalonil group, suggesting that the dual inoculation alleviates the inhibitory impact of chlorothalonil on AMF colonization rate.

### Effects of microbial inoculum and chlorothalonil on soybean plant biomass

**Figure 2:**
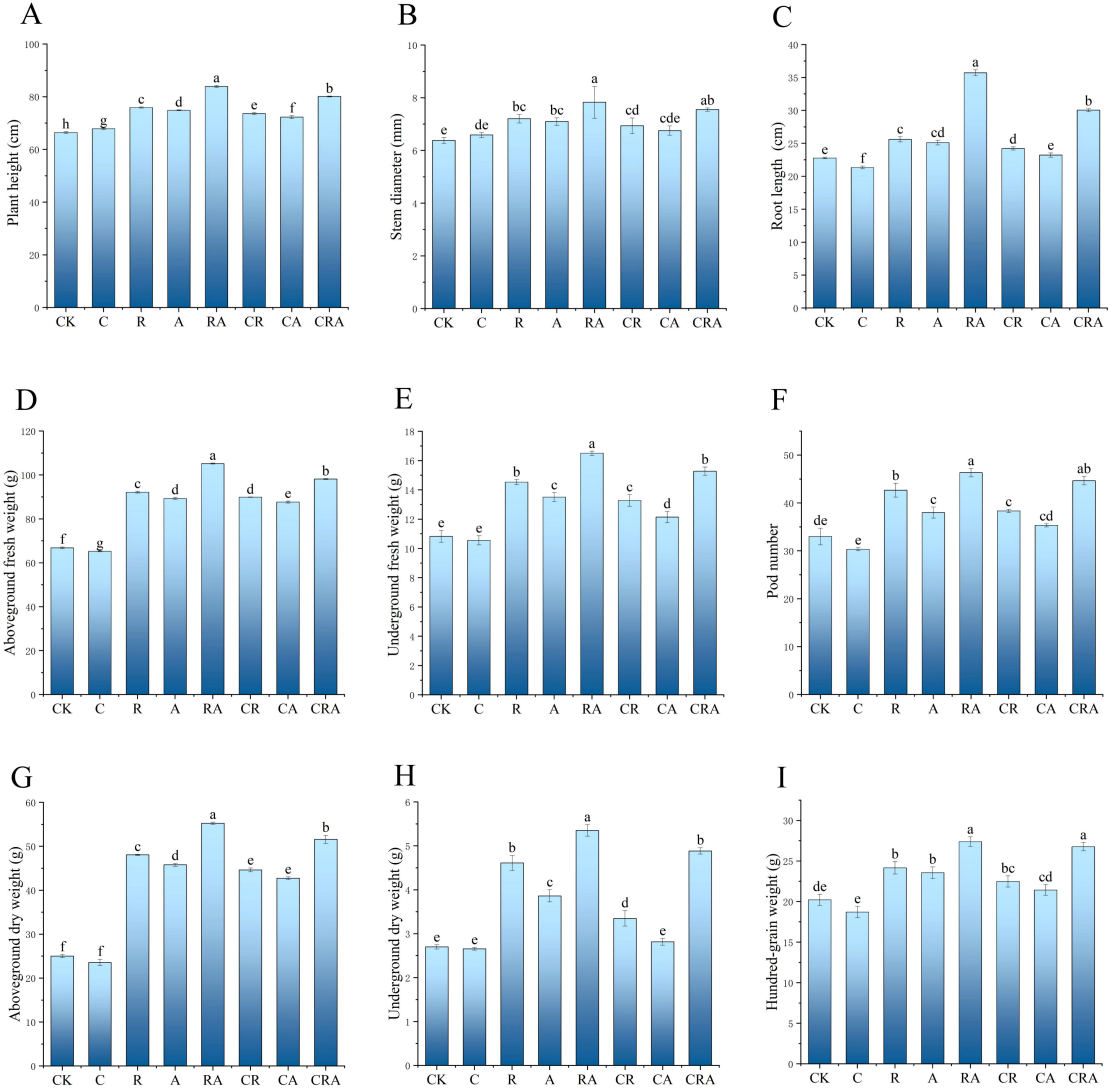
Effects of different treatments on soybean biomass. **(A)** Plant height; **(B)** stem diameter; **(C)** root length; **(D)** aboveground dry weight; **(E)** underground dry weight; **(F)** pod number; **(G)** aboveground fresh weight; **(H)** underground fresh weight; **(I)** hundred-grain weight. CK, blank control; C, chlorothalonil; R, *R. intraradices*; A, *A. calcoaceticus*; RA, *R. intraradices* and *A. calcoaceticus*; CR, chlorothalonil and *R. intraradices*; CA, chlorothalonil and *A. calcoaceticus*; CRA, chlorothalonil, *R. intraradices* and *A. calcoaceticus*.

As illustrated in **Figure 2**, the group inoculated with *R. intraradices* and *A. calcoaceticus* exhibited significantly greater values for plant height, stem diameter, root length, and hundred-grain weight compared to other treatment groups, demonstrating that the combined inoculation of *R. intraradices* and *A. calcoaceticus* significantly enhances soybean plant growth. In the chlorothalonil-treated group, plant height was marginally higher than that of the control group, while aboveground dry weight, underground dry weight, and underground fresh weight remained comparable to the control, suggesting that chlorothalonil has minimal impact on increasing soybean biomass. Furthermore, the plant height, root length, and hundred-grain weight in the *R. intraradices* and *A. calcoaceticus* inoculation group were increased by 4.72%, 18.87%, and 2.32%, respectively, compared to the group treated with both chlorothalonil and the combined microbial inoculum. This indicates that chlorothalonil, while suppressing pathogenic fungi, may also inhibit the growth of beneficial microorganisms in the rhizosphere soil, thereby negatively affecting soybean plant growth. Additionally, the plant height, stem diameter, pod number, and hundred-grain weight in the *R. intraradices* and *A. calcoaceticus* inoculation group were significantly greater than those in the groups treated with *R. intraradices* or *A. calcoaceticus*, highlighting the synergistic effect of the dual inoculation on promoting soybean biomass.

### Effects of microbial inoculum and chlorothalonil on the disease index of soybean root rot

As shown in **Figure 1C**, compared to the control group, the disease index of soybean root rot in the groups treated with *R. intraradices*, *A. calcoaceticus*, the combined inoculation of *R. intraradices* and *A. calcoaceticus*, and chlorothalonil application decreased by 66.67%, 55.67%, 77.67%, and 22.33%, respectively. These results demonstrate that both microbial inoculum and chlorothalonil contribute to the suppression of root rot occurrence, with the microbial inoculum exhibiting a more pronounced effect. Furthermore, under microbial inoculum application, the disease index of soybean root rot in the chlorothalonil-treated group was significantly lower than in the group without chlorothalonil treatment. This suggests that chlorothalonil not only eliminates pathogenic fungus but also influences the efficacy of the microbial inoculum, ultimately diminishing its ability to prevent soybean root rot. 

### Effects of microbial inoculum and chlorothalonil on nodule number

As illustrated in **Figure 1D**, the group inoculated with *R. intraradices* and *A. calcoaceticus* exhibited a significantly higher nodule number compared to other treatment groups, demonstrating that the combined inoculation of *R. intraradices* and *A. calcoaceticus* enhances the symbiotic relationship between rhizobia and soybean roots, thereby promoting nodule formation. In contrast, the nodule number in the chlorothalonil-treated group was 19.68% lower than in the control group, suggesting that chlorothalonil either suppresses rhizobial populations or inhibits the symbiotic interaction between rhizobia and soybean roots. Furthermore, the nodule number in the microbial inoculum group was significantly greater than in the non-inoculum group, indicating that *R. intraradices* and *A. calcoaceticus* mitigate the inhibitory effects of chlorothalonil on rhizobial symbiosis and facilitate the establishment of an effective symbiotic relationship between rhizobia and soybean roots.

### Effects of microbial inoculum and chlorothalonil on the total bacterial colonies in soybean rhizosphere soil

As shown in **Figure 1E**, the total bacterial colonies in the soybean rhizosphere soil were significantly higher in the microbial inoculum group compared to the control group. Specifically, the group inoculated with *R. intraradices* and *A. calcoaceticus* exhibited the highest total bacterial colonies, demonstrating that microbial inoculum enhances the soil microbial environment, improves soil fertility, and promote soybean growth and biomass accumulation. Conversely, the total bacterial colonies in the chlorothalonil-treated group were 36.28% lower than in the control group, suggesting that chlorothalonil suppresses the growth and reproduction of soil bacteria, thereby reducing bacterial populations. However, under chlorothalonil application, the total bacterial colonies in the microbial inoculum group increased significantly, with the *R. intraradices* and *A. calcoaceticus* inoculation group showing the highest total bacterial colonies. This indicates that microbial inoculum mitigates the inhibitory effects of chlorothalonil on soil bacteria, while *R. intraradices* and *A. calcoaceticus* enhance bacterial tolerance to chlorothalonil, facilitate bacterial growth and reproduction, maintain bacterial community structure.

### Effects of microbial inoculum on chlorothalonil residue in soybean rhizosphere soil and soybean grains

As illustrated in **Figure 1F** chlorothalonil was detected in the soybean rhizosphere soil of all groups treated with chlorothalonil, demonstrating its persistence and low degradation rate in the soil. Under chlorothalonil application, the residue levels of chlorothalonil in the microbial inoculum group were significantly lower than in the non-microbial inoculum group. Notably, the group inoculated with *R. intraradices* and *A. calcoaceticus* exhibited the lowest chlorothalonil residue, suggesting that microbial inoculum enhances the degradation or transformation of chlorothalonil in the rhizosphere soil. Furthermore, chlorothalonil residue was also detected in the soybean grains of all chlorothalonil-treated groups (**Figure 1F**), indicating its translocation and persistence in plant tissues. The pattern of chlorothalonil residue in soybean grains mirrored that observed in the rhizosphere soil, with the *R. intraradices* and *A. calcoaceticus* inoculation group showing the lowest residue levels. These findings suggested that microbial inoculum, particularly the combined application of *R. intraradices* and *A. calcoaceticus*, effectively reduce chlorothalonil residue in both the rhizosphere soil and soybean grains, highlighting its potential to mitigate pesticide accumulation in agricultural systems. 

### Composition of the rhizosphere bacterial community

**Figure 3:**
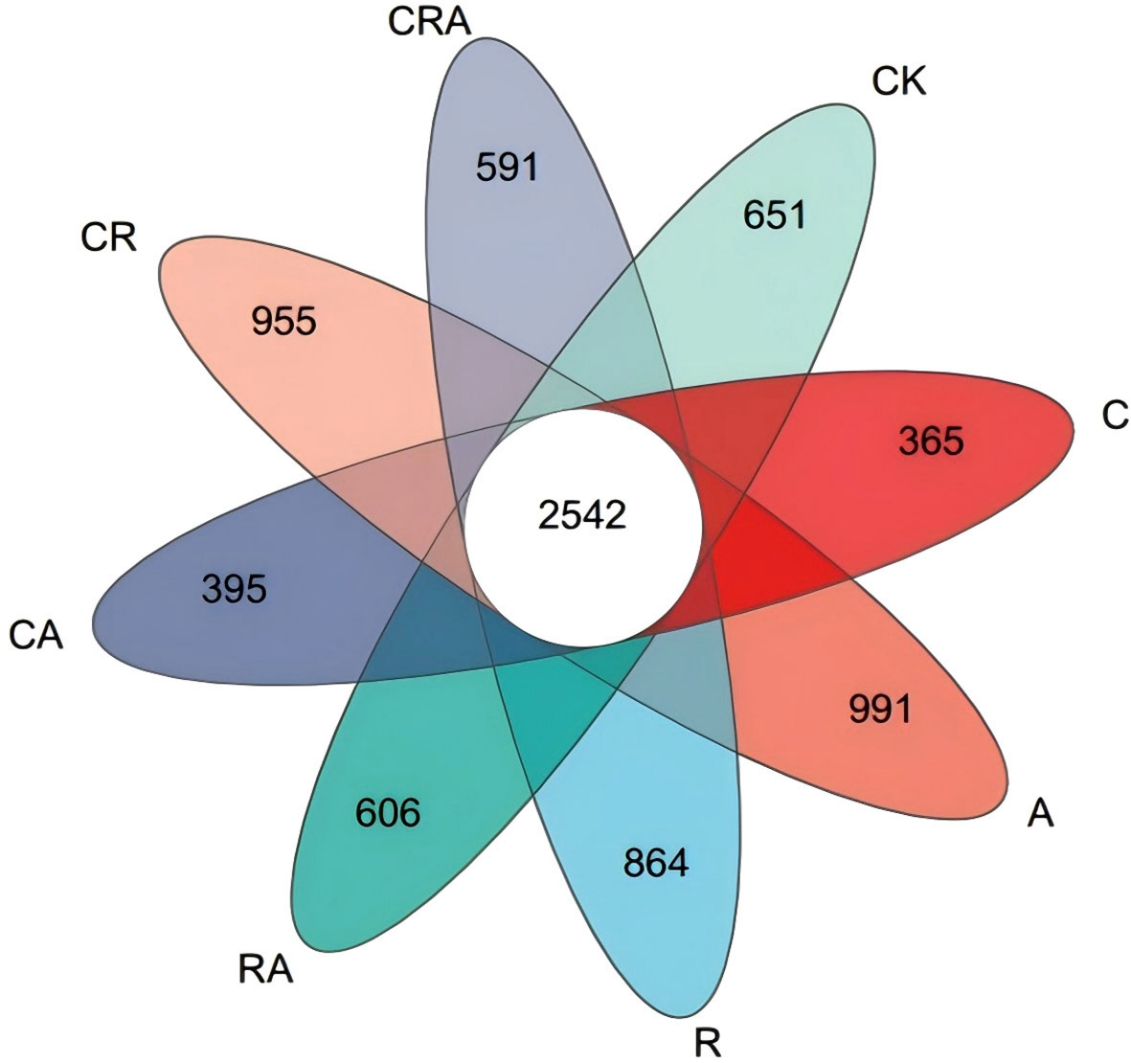
The VENN diagrams of the bacterial community composition in soybean rhizosphere soil. CK, blank control; C, chlorothalonil; R, *R. intraradices*; A, *A. calcoaceticus*; RA, *R. intraradices* and *A. calcoaceticus*; CR, chlorothalonil and *R. intraradices*; CA, chlorothalonil and *A. calcoaceticus*; CRA, chlorothalonil, *R. intraradices* and *A. calcoaceticus*.

**Figure 4:**
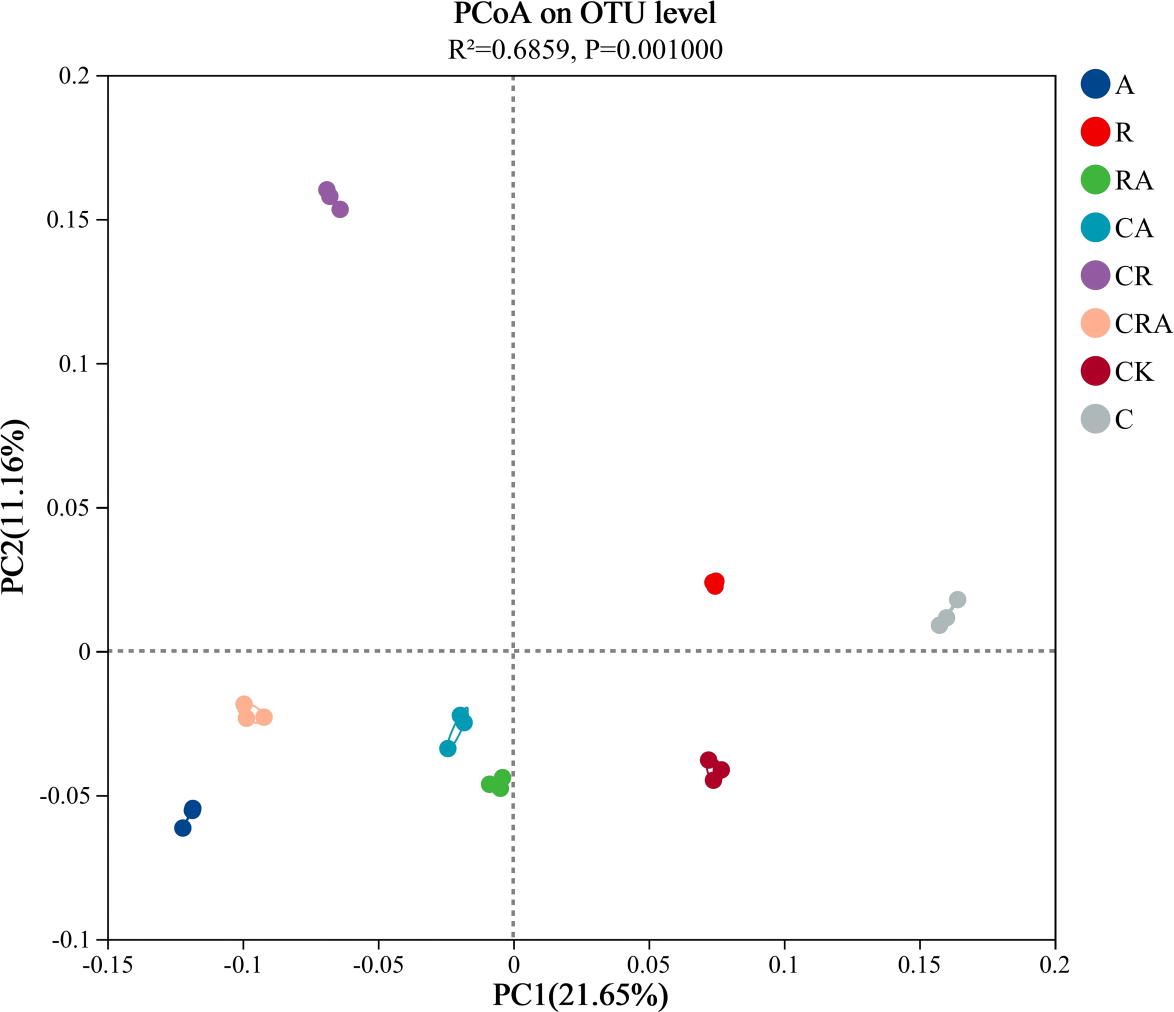
Principal coordinates analysis (PCoA) of the bacterial community composition in soybean rhizosphere soil was obtained by Bary-Curtis distance algorithm. CK, blank control; C, chlorothalonil; R, *R. intraradices*; A, *A. calcoaceticus*; RA, *R. intraradices* and *A. calcoaceticus*; CR, chlorothalonil and *R. intraradices*; CA, chlorothalonil and *A. calcoaceticus*; CRA, chlorothalonil, *R. intraradices* and *A. calcoaceticus*; R-value, Significant differences within groups; P-value, Significant differences between groups; Ellipse, 95% confidence intervals.

Alpha-diversity of the bacterial community in soybean rhizosphere soil (**Table 1**). VENN diagram was constructed to visualize common and unique OTUs across the groups based on a 97% similarity threshold (**Figure 3**). It revealed 2,542 common OTUs, representing 18.81% of the total OTUs identified across all groups. Notably, the number of unique OTUs exhibited substantial variation among the groups. The chlorothalonil-treated group displayed significantly fewer unique OTUs compared to the other groups, whereas the *R. intraradices* and *A. calcoaceticus* inoculation groups demonstrated significantly higher numbers of unique OTUs than the control group. Principal Coordinate Analysis (PCoA) based on OTU-level further elucidated the differences in bacterial community composition among the treatment groups (**Figure 4**). The results indicated significant variation (*P* = 0.001) in the composition of the rhizosphere bacterial community across the groups. Distinct segregation was observed between the control group and the other treatment groups, underscoring the substantial influence of microbial inoculation and chlorothalonil application on the composition of the rhizosphere bacterial community. Additionally, significant separation was detected between groups subjected to microbial inoculation and chlorothalonil application, confirming that the composition of the rhizosphere bacterial community was markedly altered by these treatments.

**Table 1:** Alpha-diversity of the bacterial community in soybean rhizosphere soil.

Treatments	Ace	Chao 1	Shannon	Simpson	Coverage
CK	2108.61 ± 118.06^abc^	2132.65 ± 40.71^ab^	6.18 ± 0.000^f^	0.0093 ± 0.0000^a^	0.9931 ± 0.0005^e^
C	2087.86 ± 12.97^c^	2110.38 ± 23.87^b^	6.34 ± 0.023^e^	0.0049 ± 0.0001^d^	0.9936 ± 0.0003^d^
R	2099.81 ± 33.94^bc^	2119.11 ± 6.94^b^	6.38 ± 0.015^d^	0.0051 ± 0.0002^cd^	0.9937 ± 0.0000^d^
A	2118.62 ± 3.00^ab^	2130.55 ± 7.15^ab^	6.47 ± 0.003^c^	0.0052 ± 0.0000^c^	0.9945 ± 0.0000^a^
RA	2097.00 ± 7.89^bc^	2117.78 ± 15.01^b^	6.37 ± 0.007^d^	0.0056 ± 0.0000^b^	0.9939 ± 0.0003^cd^
CR	2133.45 ± 10.54^a^	2160.24 ± 16.38^a^	6.55 ± 0.002^a^	0.0041 ± 0.0000^f^	0.9944 ± 0.0003^ab^
CA	2129.02 ± 14.35^a^	2148.54 ± 21.82^ab^	6.49 ± 0.008^b^	0.0040 ± 0.0000^f^	0.9942 ± 0.0001^abc^
CRA	2116.37 ± 1.96^abc^	2135.46 ± 3.96^ab^	6.48 ± 0.002^bc^	0.0047 ± 0.0000^e^	0.9939 ± 0.0000^bcd^

CK, blank control; C, chlorothalonil; R, *R. intraradices*; A, *A. calcoaceticus*; RA, *R. intraradices* and *A. calcoaceticus*; CR, chlorothalonil and *R. intraradices*; CA, chlorothalonil and *A. calcoaceticus*; CRA, chlorothalonil, *R. intraradices* and *A. calcoaceticus*. Values are means ± standard deviation with three replicates. Different letters indicate significant differences from different treatments (*P*< 0.05).

**Figure 5:**
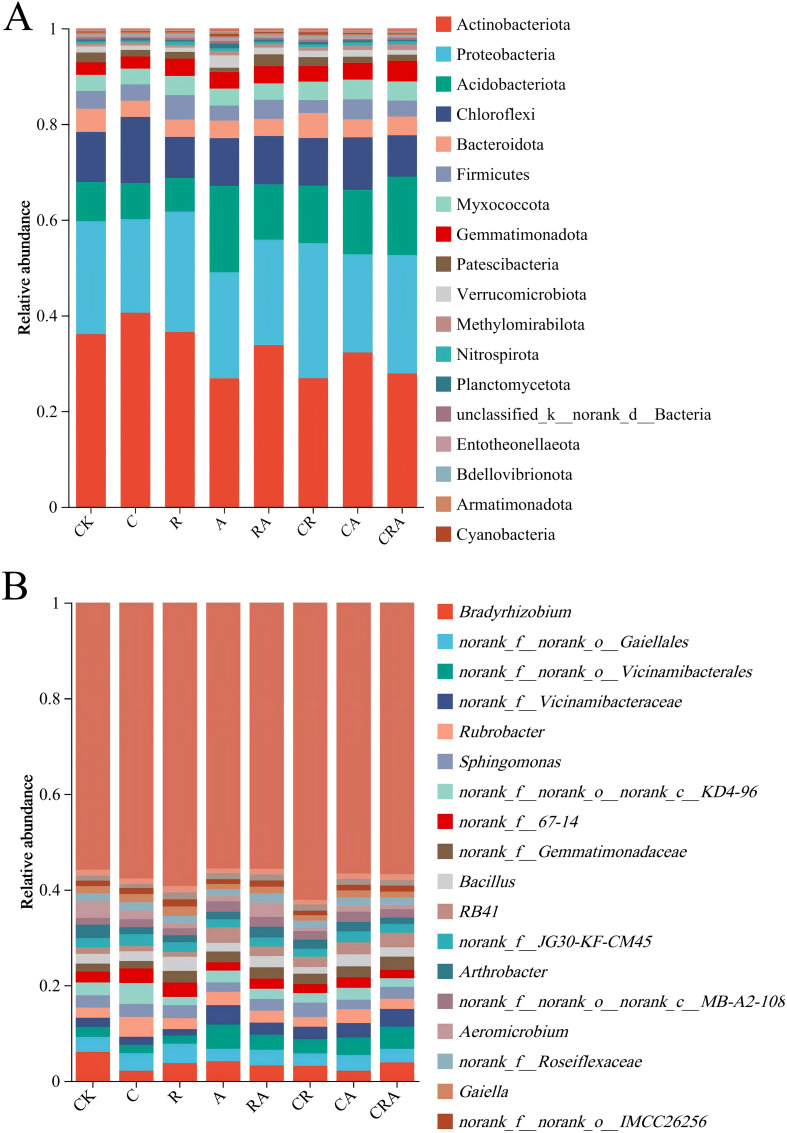
The composition of soybean rhizosphere soil bacterial community at the **(A)** phylum and **(B)** genus levels. CK, blank control; C, chlorothalonil; R, *R. intraradices*; A, *A. calcoaceticus*; RA, *R. intraradices* and *A. calcoaceticus*; CR, chlorothalonil and *R. intraradices*; CA, chlorothalonil and *A. calcoaceticus*; CRA, chlorothalonil, *R. intraradices* and *A. calcoaceticus*.

The distribution of bacterial community composition in soybean rhizosphere soil at the phylum level is illustrated in **Figure 5A**. Significant differences were observed in the relative abundance of bacterial community across the eight soil samples, with Actinobacteria, Proteobacteria, Acidobacteriota, and Chloroflexi emerging as the most dominant phyla in the experimental groups. Compared to the control group, the chlorothalonil-treated group exhibited an increase in the relative abundance of Actinobacteria and Chloroflexi by 4.48% and 3.37%, respectively, while the relative abundance of Proteobacteria and Bacteroidota decreased by 4.08% and 1.43%, respectively. In the *A. calcoaceticus* inoculation group, the relative abundances of Acidobacteriota, Gemmatimonadota, and Verrucomicrobiota were significantly higher than those in the control group. Similarly, the *R. intraradices* inoculation group demonstrated an increase in the relative abundance of Proteobacteria and Firmicutes by 1.25% and 1.41%, respectively, whereas the relative abundance of Bacteroidota decreased by 1.23% compared to the control group. Furthermore, the chlorothalonil-treated group showed significantly lower relative abundances of Proteobacteria, Acidobacteriota, Gemmatimonadota, and Methylomirabilota compared to the group treated with a combination of chlorothalonil and microbial inoculation (*R. intraradices* and *A. calcoaceticus*). These findings underscore the distinct impacts of chlorothalonil application and microbial inoculation on the composition of the rhizosphere bacterial community in phylum-level.

As illustrated in **Figure 5B**, *Bradyrhizobium* represented the most dominant genus in the control group but declined to the seventh most dominant genus in the chlorothalonil-treated group, demonstrating that chlorothalonil application significantly reduced its relative abundance. In the group treated with both chlorothalonil and microbial inoculation (*R. intraradices* and *A. calcoaceticus*), the relative abundance of *Bradyrhizobium* increased by 1.75%; However, it remained lower than that observed in the control group. In the *R. intraradices* and *A. calcoaceticus* inoculation group, *Bradyrhizobium* was identified as the second most dominant genus. Furthermore, *norank_f:norank_o:Gaiellales* (4.09%) was the most dominant bacterial genus in the *R. intraradices* inoculation group. *norank_f:norank_o:Vicinamibacterales* (5.09%) was the most dominant bacterial genus in the *A. calcoaceticus* inoculation group. Additionally, in the *R. intraradices* and *A. calcoaceticus* inoculation group, the relative abundances of *Bradyrhizobium* (3.23%), *norank_f:norank_o:Gaiellales* (3.27%), *norank_f:norank_o:Vicinamibacterales* (3.14%), and *Aeromicrobium* (3.04%) were approximately the same. These findings highlight the significant effects of chlorothalonil and microbial inoculation on the composition of the rhizosphere bacterial community.

**Figure 6:**
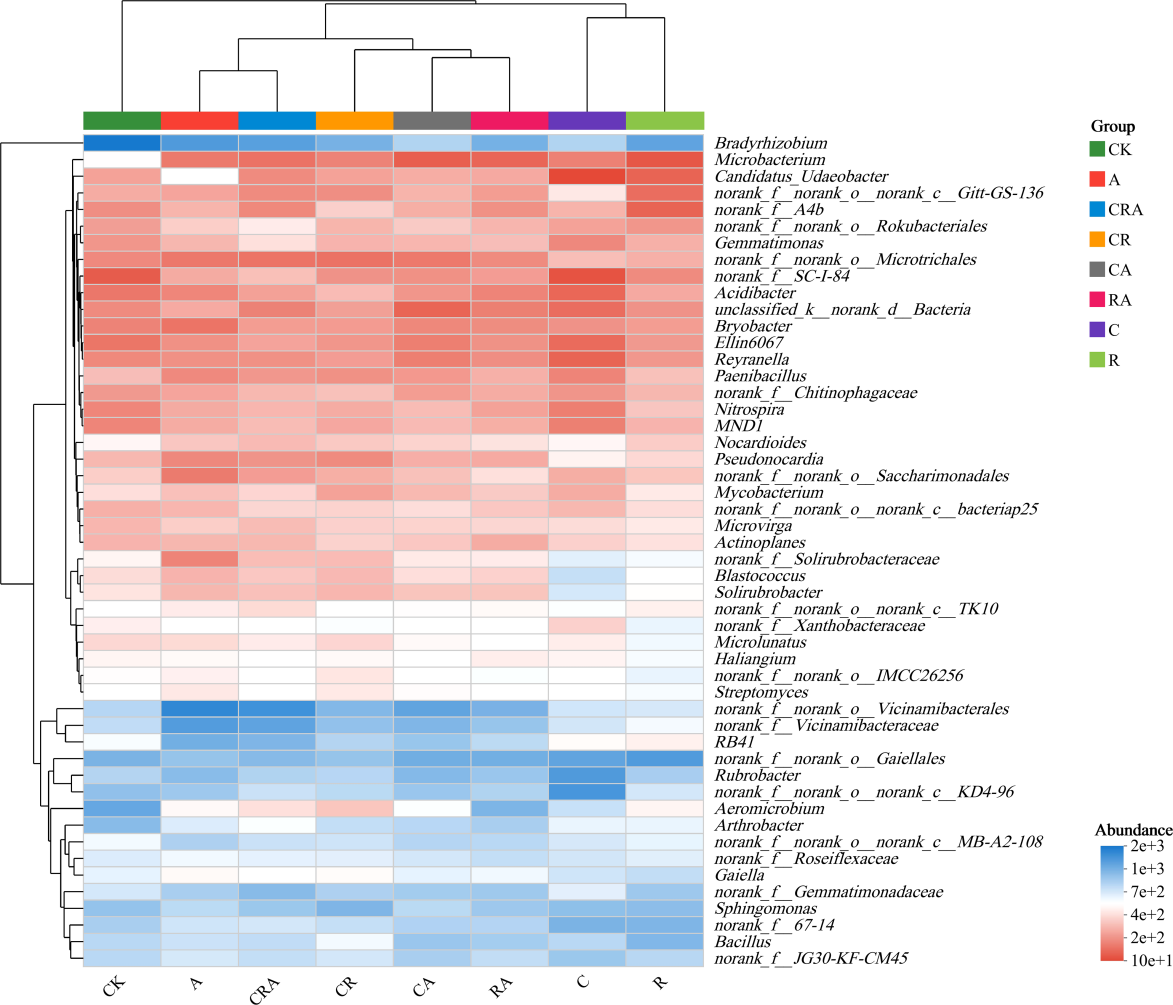
Heatmap of the 50 most abundant soybean rhizosphere soil bacterial genera. CK, blank control; C, chlorothalonil; R, *R. intraradices*; A, *A. calcoaceticus*; RA, *R. intraradices* and *A. calcoaceticus*; CR, chlorothalonil and *R. intraradices*; CA, chlorothalonil and *A. calcoaceticus*; CRA, chlorothalonil, *R. intraradices* and *A. calcoaceticus*.

Based on the top 50 genera, the eight soybean rhizosphere soil samples were clustered into three distinct groups (**Figure 6**). The blank control group displayed an independent clustering pattern, distinct from the other soybean rhizosphere soil samples. The group inoculated with *R. intraradices* and the group treated with chlorothalonil formed a separate cluster, indicating a similar bacterial community composition between these two groups. The remaining five soybean rhizosphere soil samples clustered together, suggesting a shared core function in shaping bacterial community structure. Furthermore, the analysis revealed that while the composition of the rhizosphere bacterial community exhibited similarities, the relative abundance of specific taxa was significantly influenced by inoculant and chlorothalonil treatment. These findings align with the results of the aforementioned analyses at the phylum and genus taxonomic levels, reinforcing the consistency of the observed patterns in the distribution of bacterial community composition.

## Discussion

This research systematically evaluated the interactive effects of *R. intraradices*, *A. calcoaceticus* and chlorothalonil on AMF spore density, AMF colonization rates, soybean biomass, disease index of soybean root rot, chlorothalonil residue, and bacterial community composition in soybean rhizosphere soil. The results demonstrated that AMF spore density and AMF colonization rates in microbial inoculum-treated plants were significantly elevated compared to control group (**Figure 1A, B**). Hussain et al. (2021) also reported that inoculating AMF and phosphorus-solubilizing bacteria (PSB) into the soil around corn roots can significantly increase both the AMF spore density and AMF colonization rates. These results suggested the successful establishment of symbiotic associations between *R. intraradices* and soybean roots within rhizospheric environments. Notably, *A. calcoaceticus* exhibited a promotive effect on AMF colonization rate, with dual inoculation of *R. intraradices* and *A. calcoaceticus* achieving peak mycorrhizal colonization rate, consistent with prior observations by Jie et al. (2023). *A. calcoaceticus* induces flavonoid exudation in crops and enhances AMF colonization through biochemical signaling pathways (Ordoñez et al., 2016). Comparative analysis revealed significantly depressed AMF spore density and AMF colonization rate in application of chlorothalonil groups relative to control groups, implying potential fungistatic effects of chlorothalonil on mycorrhizal proliferation. This phenomenon may derive from the chlorothalonil’s documented biocidal activity against fungal propagules, which could compromise AMF functionality through direct sporicidal action (Van-Scoy and Tjeerdema, 2014).

Soybean biomass parameters (including plant height, stem diameter, root length, and hundred-grain weight) showed significant enhancement in inoculum-treated plants versus control group, consistent with Liu et al. (2021) and Řezáčová et al. (2023) PSB enhances nutrient uptake, root development, and crop yield by increasing phosphorus availability and producing plant hormones that affect nutrient uptake and photosynthesis (Amanullah et al., 2025). Similarly, AMF networks establish symbiotic interactions that amplify rhizospheric nutrient acquisition efficiency (Pan et al., 2020). These networks overcome root morphological constraints by developing expansive functional absorptive surfaces, ultimately driving optimized soil nutrient utilization and accelerated host plant development (Xu et al., 2022). This growth promotion likely stems from complementary mechanisms: *A. calcoaceticus*-mediated solubilization of recalcitrant soil phosphates coupled with AMF hyphal networks facilitating nutrient translocation via established symbiotic interfaces (Han and Eom, 2022; Rouphael et al., 2015).

The soybean root rot disease index was significantly higher than that of the other groups, demonstrating suppression by chlorothalonil application or microbial inoculation. Notably, chlorothalonil-treated group showed higher disease index than microbial inoculation groups, indicating biological control is more effective than chemical control, consistent with Jie et al. (2025). This superior efficacy arises from fundamental mechanistic differences: chlorothalonil disrupts fungal metabolism, whereas microbial inoculants suppress pathogens via competitive exclusion and antimicrobial productio (Droby et al., 2016; Dukare et al., 2019; Sane and Mehta, 2015). Additionally, rhizobia, forming symbiotic relationships with soybeans, significantly promote plant growth and enhance soil fertility (Rutten and Poole, 2019). As depicted in **Figure 1D**, the nodule number in the chlorothalonil treatment group were lower than that in the control group, suggesting that the application of chlorothalonil may exert an inhibitory effect on nodule formation in soybean. Conversely, the nodule number in the microbial inoculum group were significantly higher than in the control group, demonstrating that microbial inoculation positively influences the establishment of root nodules. Prathima et al. (2024) demonstrated that microbial inoculation increased both the nodule number and seed yield of cowpea.

Furthermore, compared to the control group, the total bacterial colonies in the rhizosphere soil of the *R. intraradices* and *A. calcoaceticus* inoculation group exhibited an increase, with the highest total bacterial colonies observed in the co-inoculation treatment group (Etesami et al., 2021; Zhang et al., 2016). This phenomenon may be attributed to the enhanced soil fertility associated with the microbial activity of *R. intraradices* and *A. calcoaceticus*. In contrast, the total bacterial colonies in the chlorothalonil treatment group was significantly reduced compared to the control group, indicating that chlorothalonil not only suppresses pathogenic bacteria but also adversely affects the growth and proliferation of beneficial soil microorganisms. Baćmaga et al. (2018) reported that chlorothalonil can affect the microbial diversity, soil physiological and biochemical characteristics and inhibit the growth of specific microbial species. The primary active component of chlorothalonil, tetrachlorodibenzonitrile, is an organochlorine pesticide that may disrupt soil biological activity and ecological balance (Ren et al., 2024). Chlorothalonil residue were detected in both the rhizosphere soil and soybean grains of the chlorothalonil treatment groups (**Figure 1F**). Rodríguez-Seijo et al. (2025) reported that only 5-15% of the pesticides applied were used to kill the target organisms. The remaining pesticides remained in the soil, plants, and water bodies, etc. Zhao et al. (2025) demonstrated that microbial communities synergistically enhance pesticide degradation through complementary metabolic pathways, particularly in environments with complex pesticide mixtures. In this research, the chlorothalonil residue in the rhizosphere soil and soybean grains of the chlorothalonil-treated and inoculated with *R. intraradices* and *A. calcoaceticus* group was significantly lower than that in the chlorothalonil-treated group. These findings align with previous observations, indicating that microbial biodegradation can effectively reduce pesticide residue in soybean rhizosphere soil and grains, thereby mitigating the adverse impacts of pesticides on soil ecosystems and human health (Jie et al., 2023).

The composition of the bacterial community in soil constitutes a critical determinant of soil ecosystem functionality, serving as a fundamental indicator of soil quality and playing a pivotal role in maintaining ecological equilibrium (Chen et al., 2019; He et al., 2023). In this research, the dominant bacterial phyla were Actinobacteriota, Proteobacteria and Acidobacteriota in all rhizosphere soil samples, consistent with our previous findings (Jie et al., 2022, 2019). Since the soil used in this study was all sourced from the field trial soil used in our previous research, these research results indicated that the microbial inoculum might have a long-term impact on the composition of soil bacterial community and could be used as an indicator for improving soil health. Furthermore, while the composition of dominant bacterial phyla remained stable across treatments, their relative abundances varied significantly. Specifically, compared to the control group, the relative abundance of Chloroflexi in the chlorothalonil treatment group increased by 3.37%. Chloroflexi are microorganisms capable of deriving energy through the reduction of dehalogenated organochlorides, suggesting that chlorothalonil may serve as an energy substrate for these bacteria, thereby enhancing their relative abundance in the soybean rhizosphere soil (Hugenholtz et al., 2004). In contrast, the relative abundances of Acidobacteriota and Verrucomicrobiota in the *A. calcoaceticus* inoculation group increased by 9.84% and 1.33%, respectively, compared to the control group. This indicates that *A. calcoaceticus* may exert a promotive effect on the proliferation of these bacterial phyla in the rhizosphere soil. Acidobacteriota are known to play a crucial role in the soil carbon cycle, facilitating the decomposition of complex organic matter and promoting the conversion and sequestration of soil organic carbon (Gonçalves et al., 2024). Similarly, Verrucomicrobiota are closely associated with soil nitrogen cycling, particularly in the process of ammonia oxidation, thereby influencing the transformation and utilization of soil nitrogen (Flieder et al., 2021). At the genus level, the relative abundances of bacterial taxa varied significantly among treatment groups. Notably, the relative abundance of *Rubrobacter* in the chlorothalonil treatment group increased by 2.01%, while those of *Bradyrhizobium* and *Aeromicrobium* decreased by 3.96% and 1.35%, respectively, compared to the control group. *Rubrobacter* is known for its tolerance to extreme environmental conditions, whereas *Bradyrhizobium* and *Aeromicrobium* appear to be more sensitive to alterations in the soil environment induced by chlorothalonil application, resulting in their diminished relative abundances (Freed et al., 2019). In future research, we will delve into the interaction mechanism between *R. intraradices* and *A. calcoaceticus* and its impact on soil ecosystem health and agricultural productivity over time and across cropping seasons.

## Conclusions

This study demonstrates that chlorothalonil treatment reduces AMF spore density, colonization rate and bacterial colonies, whereas inoculating *R. intraradices* and *A. calcoaceticus* enhances all three parameters. Plant height and stem diameter in the *R. intraradices* and *A. calcoaceticus* inoculation group were increased by 26.37% and 22.72%, respectively, compared to the control group. Compared with the chlorothalonil-treated group, the disease index of soybean root rot in the *R. intraradices* and *A. calcoaceticus* inoculation group decreased by 71.43%. Chlorothalonil residue in both the rhizosphere soil and soybean grains in the chlorothalonil-treated and inoculated with *R. intraradices* and *A. calcoaceticus* group were decreased by 82.02% and 81.65%, respectively, compared to the chlorothalonil-treated group. These findings underscore the functional role of AMF and the potential of beneficial bacteria like *A. calcoaceticus* to improve soil health and plant growth. Furthermore, microbial inoculation enhances rhizosphere microbial diversity and modulates bacterial community composition. This research provides a theoretical basis for the biological control of soybean root rot and highlights the ecological benefits of microbial interventions in sustainable agriculture.

## Data Availability

The datasets presented in this study can be found in online repositories. The names of the repository/repositories and accession number(s) can be found below: https://www.ncbi.nlm.nih.gov/, PRJNA1228313.
